# Bioinformatics Analysis of Oral, Vaginal, and Rectal Microbial Profiles during Pregnancy: A Pilot Study on the Bacterial Co-Residence in Pregnant Women

**DOI:** 10.3390/microorganisms9051027

**Published:** 2021-05-11

**Authors:** Megumi Fudaba, Tomonori Kamiya, Daisuke Tachibana, Masayasu Koyama, Naoko Ohtani

**Affiliations:** 1 Department of Obstetrics and Gynecology, Graduate School of Medicine, Osaka City University, Osaka 545-8585, Japan; mecch1n@outlook.jp (M.F.); m1159899@med.osaka-cu.ac.jp (D.T.); masayasukoyama@gmail.com (M.K.); 2 Department of Pathophysiology, Graduate School of Medicine, Osaka City University, Osaka 545-8585, Japan; kamiya.tomonori@med.osaka-cu.ac.jp; 3 AMED-CREST, AMED, Japan Agency for Medical Research and Development, Tokyo 100-0004, Japan

**Keywords:** pregnancy, oral, vaginal and rectal microbiota, bioinformatics analysis, threatened preterm labor, preterm birth

## Abstract

Preterm birth (PTB) and threatened preterm labor (TPL), an important pre-PTB state, are major obstetric complications during pregnancy. However, their triggers have not been fully elucidated. The vagina is dominated by *Lactobacillus* species (categorized as community state types; CSTs I, II, III, and V) or by mixed anaerobes (CST IV). An abundance of the latter is associated with bacterial vaginosis (BV) and BV-triggered PTB/TPL. To identify factors that influence the diversity of vaginal microbiota associated with BV and CST IV (BV-type) bacterial profile, we performed a bioinformatic analysis of the microbial taxa using 16S rRNA amplicon sequencing data of bacterial genome in oral, vaginal, and rectal samples collected from 58 pregnant Japanese women. Interestingly, common residence of BV-associated bacteria in the vagina and rectum was individually detected in the CST IV (non-*Lactobacillus* dominated) group by species-level Spearman correlation coefficient analysis, suggesting that the rectum acts as a reservoir of BV-associated bacterial species in the CST IV group. The current study provides evidence of bacterial co-residence in vagina and rectum in the non-*Lactobacillus* dominated group, which could be targeted to reduce the risk of preterm incidence in pregnancy.

## 1. Introduction

Preterm disorders during pregnancy, such as threatened preterm labor (TPL) and preterm birth (PTB), are frequent obstetrical complications. PTB occurs in 5–15% of all pregnancies worldwide and is a leading cause of neonatal morbidity and mortality [1]. TPL is reported as a cause of PTB in 45% of cases, with other causes of preterm premature rupture of membranes occurring in 25% and maternal or fetal infections occurring in 30% of cases [2], indicating that TPL could be the major cause of PTB. Preterm infants often develop various complications, including respiratory distress, intraventricular hemorrhage, and cerebral palsy [3]. However, effective preventative strategies for TPL and PTB have not been established. While genetic background is known to influence gestation length [4,5,6,7], microenvironmental factors in the reproductive tract, including the vaginal microbiota, considerably contribute to TPL and PTB. Previous studies showed that bacterial vaginosis (BV), wherein microbial communities are dominated by non-*Lactobacillus* species, is one of the leading PTB risk factors [8,9,10]. Preventing TPL and PTB by administering antibiotics for the treatment of BV has been attempted, but its effectiveness is minimal [8,9,11,12,13,14]. Moreover, the recurrence rates of BV are reportedly high after antibiotics treatment [15,16], implying that BV-associated bacterial species might reside in other organs and eventually invade the vagina.

The female vaginal microbiota is classified into five main community state types (CSTs). It is mainly dominated by four types of homogeneous *Lactobacillus* species, categorized into the vaginal microbiome community state types (CSTs) I, II, III, and V [17]. CST IV comprises mixed anaerobes found in bacterial vaginosis (BV) patients [18]. *Lactobacillus*-dominated microbiome has long been considered the hallmark of health in the female reproductive tract. Normally, glycogen secreted from the vaginal wall plays a dominant role in *Lactobacillus* growth. Moreover, hydrogen peroxide and lactic acid, fermentation products of *Lactobacillus* species that keep the vaginal environment acidic (around pH 4.0) [17], protect the lower reproductive tract from ascending infection, and thus maintain vaginal homeostasis. Therefore, reduced *Lactobacillus* species and increased microbial diversity in the vagina are correlated with the invasion of non-*Lactobacillus* species through the lower reproductive tract.

Several analyses of human vaginal microbial profiles including a large-scale comparative racial study have been performed, wherein CST IV species was correlated with PTB in African-Americans, whereas CST I (*L. crispatus*) was negatively correlated with PTB [8,9,19]. Moreover, recent works reported bacterial infections during pregnancy that could influence maternal status and pregnancy outcomes [20,21,22]. However, simple vaginal bacterial profiling does not fully elucidate the mechanism of how vaginal microbes could cause or prevent TPL and PTB or BV-associated diseases, since other commensal microbes, such as oral or gut microbes, could influence pregnancy outcomes [2,23]. Indeed, a similarity of profiles has been observed between the oral and placental microbiota [24], raising an issue of periodontal microbes influencing the maintenance of pregnancy [2,25,26,27], although several recent studies claim that the placenta is indeed sterile [28,29,30]. In addition, the association of intestinal microbiota with BV determined by PCR analysis has also been reported [31,32], suggesting that the microbiome in other locations could be associated with CST IV, where BV-associated TPL and PTB are evident [19].

To investigate whether the increased species diversity of vaginal microbiota could originate from the commensal microbiota of other organs, we simultaneously collected saliva, vaginal fluid, and stool samples from 58 pregnant Japanese female pregnant participants and determined the bacterial profiles of the collected samples from these three organs by 16SrRNA gene sequencing. We performed QIIME2-based bioinformatics analysis at the genus and species levels and elucidated the co-residence of specific microbial species in the vagina and other organs.

## 2. Patients and Methods

### 2.1. Ethics

The studies involving human participants were reviewed and approved by the Ethics Committee of Osaka City University (Approval number 4123, date of approval: 27 December 2018) according to the Declaration of Helsinki.

### 2.2. Patients

This study was conducted at the Department of Obstetrics, Osaka City University, Osaka, Japan between September 2018 and March 2020. Fifty-eight singleton pregnant women regardless of TPL or PTB were enrolled. The inclusion criteria included: (i) singleton pregnancy; (ii) pregnant women who received perinatal management at our hospital. The exclusion criteria included: (i) multiple pregnancy; (ii) fetal anomaly; (iii) fetal growth restriction. They provided written consent and delivered at our hospital. The maternal age was 21–41 years old when delivered. The samples were collected from subjects with or without perinatal complications at the time of sample collection (Table 1). The detailed information of the enrolled pregnant women is shown in Supplementary Table S2. Moreover, since BV, wherein microbial communities are dominated by non-*Lactobacillus* species, is known to be a PTB risk factor [8,9,10], the participants were divided into two groups, a vaginal *Lactobacillus*-dominated group (the LD group; CST I, II, III, and V) and a non-*Lactobacillus-*dominated group (the non-LD group; CST IV). TPL was defined as hospitalization with the presence of regular uterine contractions or shortened cervical length to less than 25 mm before the 37th week of gestation [33,34,35]. The TPL group included pregnant women who received TPL treatment and/or experienced PTB. The non-TPL group included the rest of the subjects. PTB was defined as delivery before the 37th week of gestation, with the exception of artificial PTB.

### 2.3. Vaginal, Rectal, and Oral Sample Collection

We collected vaginal fluid from the posterior vaginal fornix and stool from the rectum with dry sterile swabs, respectively. Approximately 2 mL of saliva was collected into a sterile tube from each participant at the 28th or 29th week of gestation. Each swab was suspended separately in sterile PBS solution and centrifuged at 100× *g* for 5 min at 4 °C to remove contaminants. The supernatant was then collected and centrifuged at 5000× *g* for 10 min at 4 °C. For nucleotide analysis, the samples were snap-frozen in liquid nitrogen and stored at −80 °C. Collected saliva was dispensed into four 1.5 mL tubes and processed in the same manner as the PBS-suspended vaginal fluid and stool samples. Samples were kept on ice during the procedure until frozen.

### 2.4. DNA Extraction for Next-Generation Sequencing

Bacterial DNA extraction from vaginal, rectal, and oral samples was carried out by BIKEN Biomics Inc. (Osaka, Japan) using an automated DNA extraction machine (GENE PREP STAR PI-480, Kurabo Industries Ltd., Osaka, Japan) and NR-201 DNA extraction kit (Kurabo Industries Ltd., Osaka, Japan). DNA was extracted according to manufacturer’s protocol. In brief, 0.5 g of glass beads (0.1 mm diameter, IEDA Trading Corp., Tokyo, Japan) and 300 μL of No. 10 solution (NR-10025) were added to each stool suspensions (200 μL each), following which the mixture was agitated using DISRUPTOR-GENIE (Scientific Industries Inc., New York, USA) at 3000 rpm for 90 s. After centrifugation at 9700× *g* for 5 min, the supernatant of stool samples was collected and transferred to the machine fitting strips of eight sample tubes. Next, 150 μL of No. 2 solution (NR-2025) supplemented with proteinase K (final concentration 0.4 mg/mL, FUJIFILM Wako Pure Chemical Corporation, Osaka, Japan) and 150 μL of No. 10 solution (NR-10025) were added to the sample solution and the tubes were subjected to the automated DNA extraction machine, GENE PREP STAR PI-480. The concentration of extracted DNA was determined using Qubit assays (Thermo Fisher Scientific Inc., Delaware, USA). Extracted DNA samples were stored at −30 °C until use.

### 2.5. 16S Ribosomal RNA (rRNA) Gene Amplification and Sequencing

DNA library preparation and sequencing were carried out by BIKEN Biomics Inc. (Osaka, Japan). Each DNA library was prepared according to the Illumina 16S Metagenomic Sequencing Library Preparation Guide, with 27Fmod (5′-AGRGTTTGATCMTGGCTCAG-3′) and 338R (5′-TGCTGCCTCCCGTAGGAGT-3′) primers, which target the V1–V2 hypervariable region of the bacterial 16S rRNA gene. Samples were analyzed by 251 bp paired-end sequencing using a MiSeq system (Illumina, inc., California, USA) and a MiSeq Reagent v. 2 500 cycle kit.

### 2.6. Vaginal, Fecal, and Oral Microbiota Profiling

Sequence reads were analyzed using the Quantitative Insights into Microbial Ecology 2 version 2019.4 (QIIME2) pipeline. Detailed QIIME2 commands and options are summarized in Table S1. The paired-end sequences obtained were de-noised and merged using the DADA2 R library in QIIME2. Taxonomic assignment of 16S rRNA sequences was performed using a Silva 132 99% OTU classifier. Differences in bacterial abundance were assessed via linear discriminant analysis (LDA) effect size (LEfSe), using the online Galaxy version (http://huttenhower.org/galaxy/, last accessed: 10 March 2021). Amplicon sequence variants (ASVs) were aligned using mafft software in QIIME2 and subsequently used for diversity analysis with fasttree software in QIIME2. The observed-ASVs, Shannon–Wiener indices and Pielou’s evenness indices were calculated at a sequence depth of 11,009 reads per sample, with 10 random iterations of the feature units table using QIIME2. To ensure equal sequencing depth, sample libraries were subsampled to 12,500 reads for Bray–Curtis distance and 12,426 for Weighted Unifrac distance, which were the number of reads in each library with the fewest reads. β-Diversity and principal coordinates analysis (PCoA) plots were calculated using the Bray–Curtis distance and Weighted Unifrac distance in QIIME2. Additionally, a heat map comparing samples based on the relative abundance of species-level taxa was generated via Ward’s method and hierarchical clustering using the pheatmap package v. 1.0.12 running in *R* software v4.0.3 (R Foundation for Statistical Computing, Vienna, Austria). ASVs were analyzed using National Center for Biotechnology Information (NCBI) BLASTN and the 16S ribosomal RNA sequences database for species identification (identity > 97%, coverage > 95%). If the ASVs did not match these conditions, we considered the sequences to indicate other species of each genus.

### 2.7. Statistics

Data were analyzed using the *t*-test, Chi-square test, Fisher’s exact test, Mann–Whitney U test, Kruskal–Wallis test, Spearman correlation coefficient, and Dunn’s multiple comparisons test as indicated in the text or figure legends. In all tests, *p* < 0.05 was considered statistically significant.

## 3. Results

### 3.1. Vaginal Lactobacillus Classification by 16S rRNA Gene Sequencing Analysis

**Figure 1:**
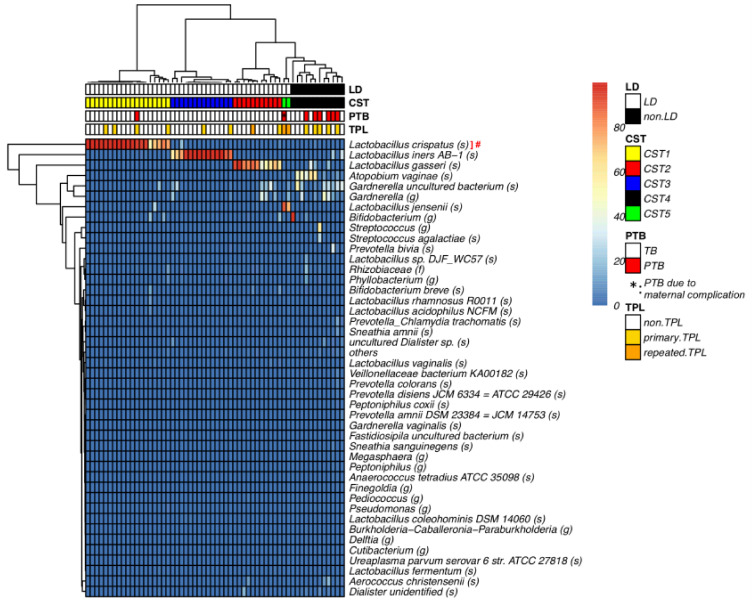
The vaginal microbiome was classified into five CSTs. Heatmap of microbial taxa found in the vaginal bacterial communities of 58 pregnant women (color key is indicated on the right bar). The taxon was defined as “others” when the maximum value of percentage was less than 1% in each vaginal sample. Ward’s method clustering of samples based on the species composition and abundance of vaginal bacterial communities that define community state types (CST) I to V on the lower bar, CSTI (*Lactobacillus crispatus*, yellow), CSTII (*L. gasseri*, red), CSTIII (*L. iners*, blue), CSTIV (mixed anaerobes, black), CSTV (*L. jensenii*, green). #: The ASVs of “*Lactobacillus* (g)” and “*Lactobacillus_Chlamydia trachomatis* (s)” were identified as genus of *Lactobacillus crispatus* by BLASTn. *: One PTB was due to maternal complication (* is shown in the small red square of PTB line at the upper part of the heat map). The raw data are available in Table S4.

First, to classify vaginal samples based on CST, 16S rRNA gene sequencing analysis was employed on vaginal fluid samples obtained from 58 pregnant women. Hierarchical clustering was performed, and Ward’s method was used to classify the vaginal microbiome into four *Lactobacillus*-dominated CSTs: CST I (*L. crispatus*), CST II (*L. gasseri*), CST III (*L. iners*), CST V (*L. jensenii*), as well as a non-*Lactobacillus*-dominated CST with mixed anaerobes, CST IV (19 in CST I, 11 in CST II, 14 in CST III, 12 in CST IV, and 2 in CST V) (Figure 1) [36]. Since the increased diversity of vaginal bacterial profile in BV is associated with the risk of PTB [8,9,10], the participants were divided into two groups according to *Lactobacillus* dominancy in the vagina: *Lactobacillus*-dominated group (the LD group; CST I, II, III, and V) and non-*Lactobacillus-*dominated group (the non-LD group; CST IV). Subject characteristics, pregnancy, and delivery outcome details are summarized in Table 1. The percentage of patients diagnosed with TPL was 21.7% in the LD group and 41.7% in the non-LD group. However, there was no statistically significant difference in TPL occurrence between the two groups. The percentage of PTB patients was 2.2% in the LD group and 41.7% in the non-LD group, indicating a significantly higher incidence rate of PTB in the non-LD group (Table 1, Table S2).

### 3.2. Bioinformatics Analysis of the Vaginal, Rectal, and Oral Microbial Profiles Using 16S rRNA Gene Amplicon Sequencing Data

**Figure 2:**
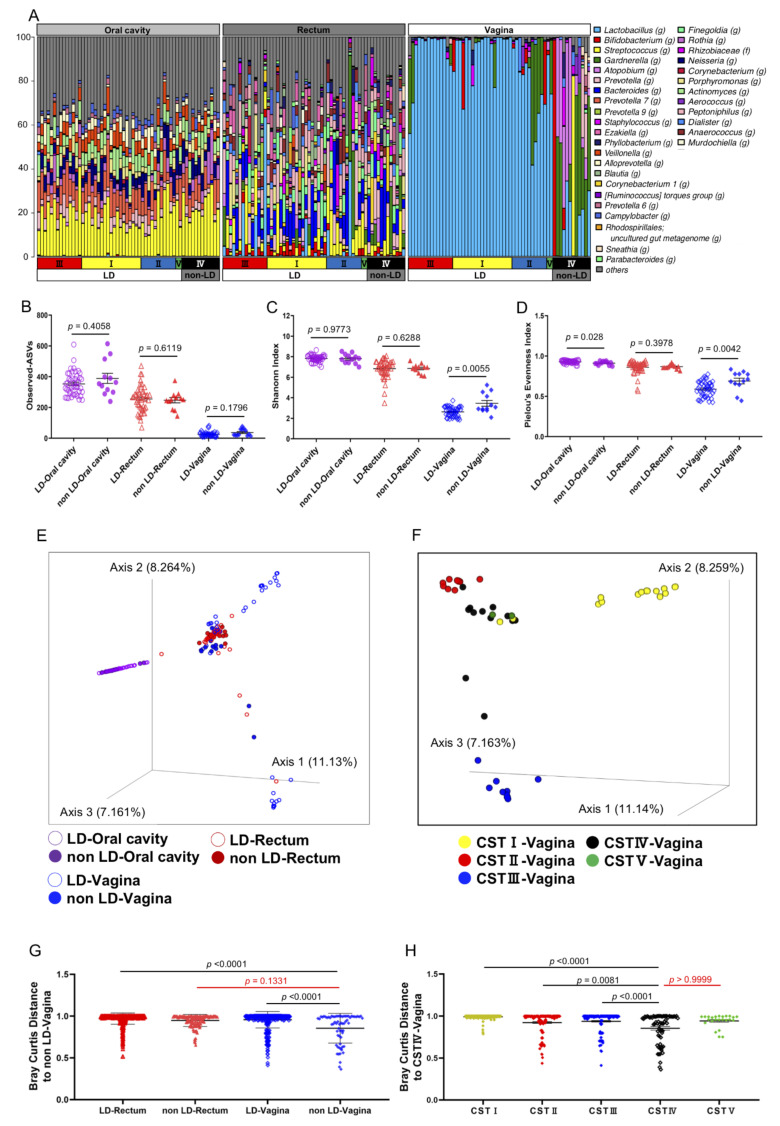
Integrated analysis of oral, vaginal, and rectal microbiota. The microbiome from the oral cavity, vagina, and rectum from 58 pregnant women including *Lactobacillus*-dominated vaginal microbe group (LD group) and non-*Lactobacillus*-dominated vaginal microbe group (non-LD group) were determined using 16S rRNA gene sequencing analysis. (**A**) The taxonomic distribution of the individual microbes is shown at the genus level. The taxon was defined as “others” if the maximum value of percentage was less than 10% in each sample. The Richness, Diversity and Evenness of samples (non-LD (*n* = 12), LD (*n* = 46)) as derived using observed ASVs (**B**), Shannon Index (**C**), Pielou’s Evenness Index (**D**), Bray–Curtis (**E**,**F**), Bray–Curtis distance to non-LD group (**G**), to CST IV-vagina (**H**), respectively. For (**B**–**D**) data show the mean ± SEM and Dunn’s multiple comparisons test was applied. For (**G**,**H**), data show the mean ± SEM and statistical significance relative to non-LD vagina group determined by Dunn’s multiple comparison test. Bray–Curtis 3D plot colored for non-LD- and LD-group across the whole cohort as bar. The Bray–Curtis was used to explore and visualize any similarities or dissimilarities in relation to non-LD and LD group with the percentage proportion of variance attributable to each axis being Axis 1 = 11.13%, Axis 2 = 8.264%, Axis 3 = 7.161% (**E**) and Axis 1 = 11.14%, Axis 2 = 8.259%, Axis 3 = 7.163% (**F**). The raw data are available in Table S4.

The presence of CST IV mixed anaerobes in the vagina is recognized as a potent trigger of TPL and PTB [8,9]. Although previous studies have suggested the co-existence of specific microbes in the rectum and vagina of BV patients using PCR analysis [31,32], a detailed bioinformatic analysis of the whole microbiome in the rectum and vagina of pregnant women has not been carried out. Moreover, oral microbiota composition was reported to be associated with adverse pregnancy outcomes [2]. Therefore, in order to investigate the similarities or the differences between the oral, vaginal, and rectal microbial profile of non-LD group (CST IV) and LD group (the other four CST groups) subjects, saliva, vaginal fluid, and stool samples simultaneously obtained from the same pregnant women were analyzed for their genus-level microbial profiles (Figure 2a). We performed bioinformatic analysis of the 16S rRNA amplicon sequencing results using QIIME2 software. The analysis of microbial α-diversity, a measure of the variation within a particular sample, was evaluated based on the number of observed amplicon sequence variants (ASVs) (Figure 2b), the Shannon Index (Figure 2c) and the Pielou’s Evenness Index (Figure 2d). As expected, the Shannon Index and the Pielou’s Evenness Index of vaginal microbiota differed significantly between the LD and non-LD group. The samples from the other locations showed no difference between the groups by the Shannon Index (Figure 2c). However, Pielou’s Evenness Index showed differences of oral samples between the LD and non-LD groups, suggesting that evenness of the bacterial species is different between these groups (Figure 2d). The β-diversity analysis, which reflects the dissimilarity of the microbial composition between groups, revealed a similar saliva microbiome distribution in the LD and non-LD groups in three-dimensional plots of PCoA calculated by Bray–Curtis distance (Figure 2e), without any bacterial overlap with those from vaginal fluid or stool. Intriguingly, however, vaginal and rectal microbiota from the non-LD group overlapped well (Figure 2e) and were not significantly different (Figure 2g, *p* = 0.1331). In contrast, the vaginal and rectal microbes from LD group subjects did not overlap (Figure 2e).

Next, we focused on the vaginal PCoA plots in Figure 2f, and re-evaluated them according to the CST classification. Interestingly, although there were only two cases, the vaginal microbiota from CST V overlapped with that of CST IV in the non-LD group (Figure 2f). Accordingly, no significant difference was observed in the Bray–Curtis distance between CST V and CST IV (Figure 2h, *p* > 0.9999). These results suggest that a certain LD subject group, CST V (*L. jensenii*) in this case, with previous TPL history (Table S2), harbored vaginal microbiota similar to that of the non-LD group (CST IV). However, since *L. jensenii* (CST V) constitutes a relatively minor share of the human vaginal microbiome [36,37,38,39], further investigation with a higher number of cases is necessary. In addition, we noticed that CST IV overlapped with three CST I members (*L. crispatus*) (Figure 2f). We examined the bacterial profiles of these three overlapping cases and found that they harbored a lesser abundance of *L. crispatus* (approximately 50–75%) compared with other non-overlapping CST I members harboring *L. crispatus* with more than 90% occupancy (Figure 2a,f). In contrast, CST III members (*L. iners*) in our study did not overlap with CST IV (Figure 2f,h).

**Figure 3:**
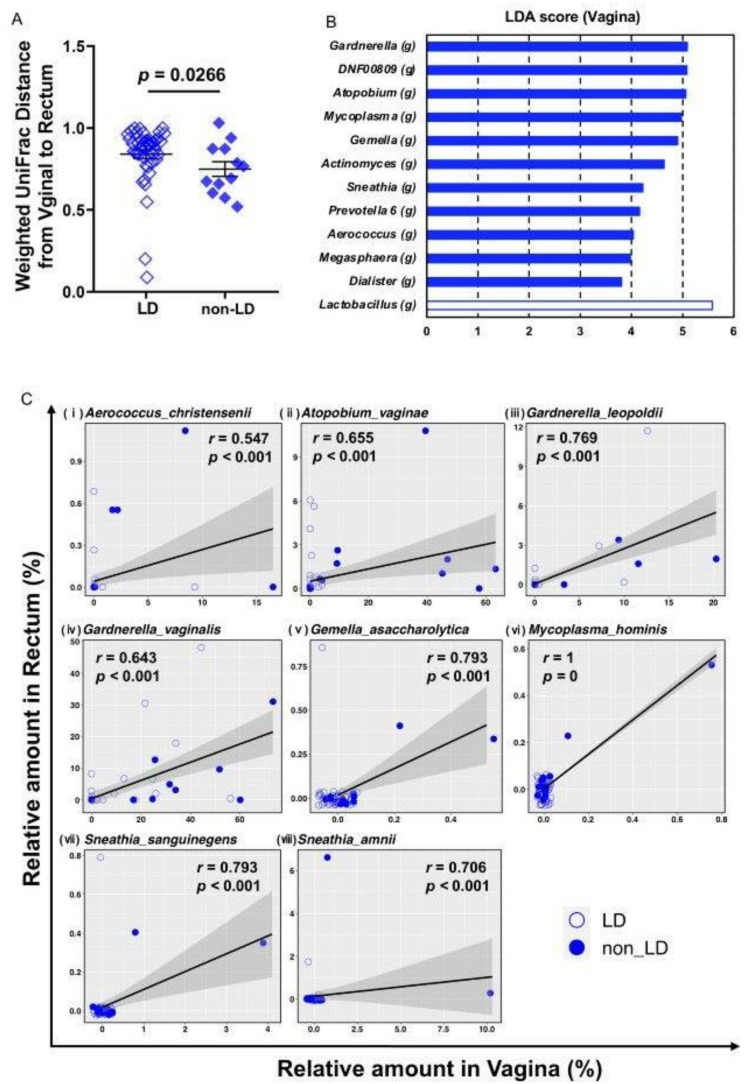
The microbial profiles of the paired vaginal and rectal samples from the same patients revealed the co-residence of specific bacterial species. (**A**) Weighted UniFrac Distance from Vagina to Rectum, data show the mean ± SEM and Mann Whitney U test was applied. (**B**) Differences in vaginal microbiota composition at the genus level were determined using the linear discriminant analysis (LDA) effect size (LEfSe) analysis (LDA score > 2). The bar graph shows the significantly different genera in the non-LD and LD group. (**C**) The correlation of their amount in vagina and rectum was examined for each species using Spearman correlation coefficient. The correlation coefficient and *p-*values are as shown in the graph. The raw data are available in Table S4.

Furthermore, we investigated the co-residence of the specific bacterial species in the vagina and rectum using paired samples. By using Weighted UniFrac Distance analysis (Figure 3a), we found that the microbial profiles of the paired vaginal and rectal samples from the same patients in the non-LD group were significantly closer than those of the samples from the patients in the LD group. LEfSe analysis of vaginal microbiota between the LD and non-LD group was performed to identify microbes that were different between groups (Figure 3b). Among the identified bacteria, we investigated whether the co-residence of these specific bacterial species was observed, both in the vagina and rectum, using paired samples. We identified several bacterial species, such as *Atopobium vaginae and Gardnerella vaginalis*, which are known to cause BV-associated TPL and PTB, residing both in the vagina and rectum in individual non-LD patients with significant correlation at the species level (Figure 3c). Some of the significant correlations in Figure 3c seem to be strongly influenced by a very few data dots when the majority of the participants do not have the indicated bacterial species in the rectum or vagina (Figure 3c v–viii). However, although very few, we think these dots also play a part of the evidence of bacterial co-residence in the individual rectum and vagina. Consistently, moreover, all the bacterial species shown in Figure 3c have been reported as causal bacterial species for BV [40,41,42,43]. These analytical results using paired samples could indicate that the microbial profiles of the vagina and rectum were more similar in the non-LD (CST IV) group than in the LD group. The BV-associated vaginal bacterial species in non-LD (CST IV) group resided in the rectum, supporting the notion that the rectum acts as a reservoir for BV-associated vaginal bacteria.

TPL and PTB were also observed in the LD group carrying a single *Lactobacillus* species. Since periodontal disease-causing bacteria are associated with PTB due to their transfer to the placenta via circulation [27], we conducted further LEfSe analysis of oral microbiota between TPL and non-TPL subjects in the LD group (Table 1). The results revealed the enrichment of significantly different genera, including the genus *Aggregatibacter*, some of which are known as a periodontal disease-causing bacteria [26], in the TPL group (Figure S1A,B, Table S3). However, our multiple-testing analysis revealed no significantly abundant oral microbiota in the TPL subjects, although further analysis is needed.

## 4. Discussion

We investigated the influence of oral and rectal microbiota on the vagina as potential predisposing factors for TPL and PTB, because the early detection of TPL and prevention of PTB are indeed crucial in pregnancy. We collected saliva, vaginal fluid, and stool samples from 58 pregnant women, and performed a bioinformatics analysis of the commensal microbial profiles in samples from these three locations of the individual pregnant women. BV is known as a major risk factor for TPL and PTB [18], and BV-causing bacterial species have been increasingly identified in the last decade [42,43,44]. Consistent with the results of a previous study [19], our results revealed that pregnant women carrying vaginal microbiota categorized as CST IV were at a high risk for both TPL and PTB (Table 1). Our bioinformatics analysis showed that the CST IV bacterial profile in the vagina and rectum clearly overlapped, and BV-associated bacterial species, such as *Atopobium vaginae and Gardnerella vaginalis,* which are known to cause TPL and PTB, indeed resided in the vagina as well as individually in the rectum (Figure 3c), suggesting that the rectum acts as a reservoir of TPL- and PTB-causing bacteria.

The administration of antibiotics targeting BV-causing bacteria in vagina has been attempted, but its effectiveness seemed to be minimal [8,9,11,12,13,14], and the recurrence rates of BV were reported to be high after antibiotics treatment [15,16]. Therefore, recently, alternative non-antibiotic approaches, such as the usage of probiotic or prebiotic products including application of *Lactobacilli*, lactic acid, and sucrose gel, combined with estriol, have been tried. Not only vaginal application of *Lactobacilli* [45,46,47], but also oral intake of *Lactobacilli* [48], showed better results than placebo, suggesting that intestinal microbiota could play an interactive role with that of the vagina.

A recent large-scale analysis of human vaginal microbes revealed that *L. crispatus* was negatively correlated with PTB [9,19]. In addition, several studies reported that some carriers of homogenous *Lactobacillus* species, particularly a higher rate with *L. iners*, experience spontaneous PTB [39], suggesting that other unknown factors could influence the onset of TPL and PTB. We, therefore, carried out oral and rectal microbial analysis in the vaginal LD group. We also investigated whether such species were present among oral microbiota, as periodontal bacteria are known to cause PTB by circulation and colonization in placenta [27], although several recent studies claim that placenta is sterile [28,29,30]. However, we did not detect a statistically significant difference in oral bacteria between the LD and non-LD group in our cases. Although the CST III group (*L. iners*), in another study, showed the increased incidence of PTB [19], CST III group in this study showed low TPL and/or PTB incidence with few divergent vaginal microbial profiles (Figure 1 and Figure 2f,h, Table S2). These results imply that environmental and tissue microenvironmental factors might influence the characteristics of vaginal bacterial species in vaginal LD group.

The limitations of this study are that we did not collect urine samples, although urinary tract infections can cause PTB [49,50,51]. In addition, fungal infection occurs in these locations, and Candida infection has been reported to be a possible trigger of PTB [52,53]. However, we did not examine the presence of fungus in this study. These perspectives need to be considered carefully for a better understanding of the factors associated with the role of BV-associated bacteria that could cause TPL and PTB.

In summary, our investigation, involving the bioinformatics analysis of the commensal microbial profile using oral, vaginal and rectal subjects from 58 pregnant Japanese women, showed evidence of bacterial co-residence in vagina and rectum in the non-LD group. Although our pilot study was performed in a single center in Osaka, Japan, the analysis of our subjects showed a similar CST type distribution to that of a previously reported large-scale study [19]. We precisely examined the specific species from individual pregnant woman and revealed that the rectum is a reservoir of the vaginal microbiota. Our findings suggest that not only the vaginal bacterial species but also those of the rectum are potential targets for improving vaginal bacterial diversity and reducing the risk of preterm incidence in pregnancy.

## 5. Conclusions

To identify factors that influence the increased diversity of vaginal microbiota associated with BV and CST IV-type bacterial profile, we performed QIIME2-based bioinformatics analysis of commensal microbial taxa of oral, vaginal, and rectal samples collected from 58 pregnant women. The common residence of BV-associated bacteria in the vagina and rectum was individually detected in the CST IV (non-LD) group at species-level, suggesting that the rectum acts as a reservoir of BV-associated bacteria in the CST IV group. The current study provides evidence of specific bacterial co-residence in vagina and rectum, and that both microbial species could be targeted to reduce the risk of preterm incidence in pregnancy.

## Figures and Tables

**Table 1:** Clinical characteristics of LD and non-LD group patients. The clinical characteristics of LD (*n* = 46) and non-LD (*n* = 12) group patients. Mann–Whitney U test was performed for continuous variables, and Fisher’s exact test was performed for the others. Threatened preterm labor was defined as hospitalization with the presence of regular uterine contractions or shortened cervical length to less than 25 mm before 37th gestational week. Preterm birth was defined as delivery before 37th gestational week, except for artificial preterm birth.

Characteristics (*n* = 58 in total)	LD (*n* = 46)	non-LD (*n* = 12)	*p* Value
Age (median)	33 (21–41)	33 (30–40)	0.87
BMI (kg/m^2^) at pre-pregnancy (median)	21.9 (16.1–32.9)	21.6 (18.1–28.4)	0.806
BMI (kg/m^2^) at delivery (median)	26.0 (19.1–34.0)	24.9 (21.0–32.4)	0.602
Body weight gain during pregnancy (median)	9.2 (−5–21.1)	9.7 (2.8–15)	0.719
Parity	18 (39.1%)	4 (33.3%)	0.493
Uterine Leiomyoma	6 (13.0%)	1 (8.3%)	1
Diabetes Mellitus	2 (4.3%)	1 (8.3%)	0.508
Gestational Diabetes Mellitus	8 (17.4%)	0	0.185
Hypertensive Disorders of Pregnancy	3 (6.5%)	1 (8.3%)	1
Threatened preterm labor	10 (21.7%)	5 (41.7%)	0.265
Premature rapture of membrane	1 (2.2%)	1 (8.3%)	0.374
Gestational age at delivery (median)	38w5d (35w6d–41w4d)	38w0d (34w0d–41w1d)	0.076
Spontaneous preterm birth	1 (2.2%)	5 (41.7%)	0.001
Birth weight of neonate (median)	2990 (2285–4035)	2772.5 (2056–3524)	0.230

## Data Availability

The datasets presented in this study can be found in the DDBJ database (DRA: https://ddbj.nig.ac.jp/DRASearch/, last accessed: 15 April 2021) under the following accession numbers. DRA (DDBJ Sequence Read Archive) accession No: SUBMISSION(DRA011283), EXPERIMENT (DRX250918—DRX251091), RUN (DRR261215—DRR261388), STUDY (DRP006831). BioProject accession No: PRJDB10581, BioSample accession NO: SAMD00264096—SAMD00264269.

## Supplementary Materials

The following are available online at https://www.mdpi.com/article/10.3390/microorganisms9051027/s1, Figure S1: Microbial profile analysis of oral cavity from the TPL and non-TPL group of LD group, Table S1: Analysis workflow and QIIME2 parameter settings used in this study, Table S2: Patients’ information, Table S3: Raw data of Figure S1, Table S4: Raw data of Figures S1–S3.
