# Supplementary material for: Bioinformatics Analysis of Oral, Vaginal, and Rectal Microbial Profiles during Pregnancy: A Pilot Study on the Bacterial Co-Residence in Pregnant Women
Source: Microorganisms. 2021 May 11;9(5):1027. doi: 10.3390/microorganisms9051027 (PMC8151423; doi:10.3390/microorganisms9051027)
Supplement: Supplementary file 1 [file microorganisms-09-01027-s001.zip › Figure S1.pdf]

A

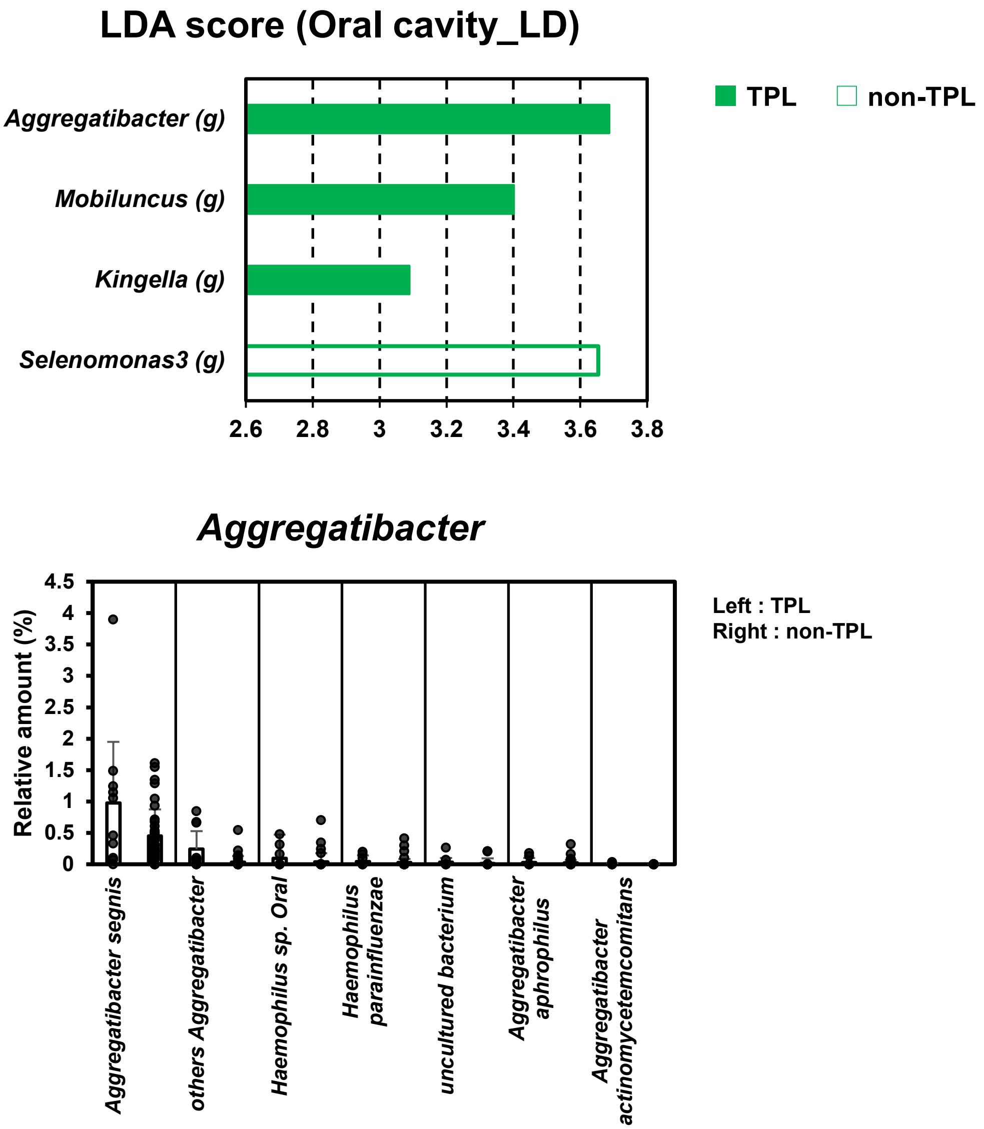

Figure S1. The significant different genera were found in oral cavity of the LD group in the TPL group.

(**A**) Differences in oral microbiota composition of the LD group at the genus level were determined using the linear discriminant analysis (LDA) effect size (LEfSe) analysis (LDA score > 2). The bar graph shows the significantly different genera in the non-TPL and TPL group. (**B**) The bar graph showing the relative abundance at the species level from *Aggregatibacter*. Data show the mean ± SEM, and Dunn's multiple comparisons test was applied. Left, TPL group; Right, non-TPL group.
