# Supplementary material for: Bioinformatics Analysis of Oral, Vaginal, and Rectal Microbial Profiles during Pregnancy: A Pilot Study on the Bacterial Co-Residence in Pregnant Women
Source: Microorganisms. 2021 May 11;9(5):1027. doi: 10.3390/microorganisms9051027 (PMC8151423; doi:10.3390/microorganisms9051027)
Supplement: Supplementary file 1 [file microorganisms-09-01027-s001.zip › Table S1 20210504.pdf]

Table S1. Analysis workflow and QIIME2 parameter settings used in this study*

QIIME2 command (A)	Options
qiime tools import	--type 'SampleData[PairedEndSequencesWithQuality]'
	--input-path manifest_XX.csv
	--output-path demux_XX.qza
	--input-format PairedEndFastqManifestPhred33
qiime demux summarize	--i-data demux_XX.qza
	--o-visualization demux.qzv
qiime dada2 denoise-paired	--i-demultiplexed-seqs demux_XX.qza
	--o-table table_XX.qza
	--o-representative-sequences rep-seqs_XX.qza
	--p-trim-left-f 0
	--p-trim-left-r 0
	--p-trunc-len-f 250
	--p-trunc-len-r 250
	--o-denoising-stats stats_XX.qza
	--p-n-threads 0
qiime metadata tabulate	--m-input-file stats_XX.qza
	--o-visualization stats_XX.qzv
qiime feature-table summarize	--i-table table_XX.qza
	--o-visualization table_XX.qzv
	--m-sample-metadata-file XX.txt
qiime feature-table tabulate-seqs	--i-data rep-seqs_XX.qza
	--o-visualization rep-seqs_XX.qzv
qiime feature-classifier classify-sklearn	--i-classifier (B).qza
	--i-reads rep-seqs_XX.qza
	--o-classification taxonomy.rep-seqs_XX.qza
	--p-n-jobs 18
qiime metadata tabulate	--m-input-file taxonomy.rep-seqs_XX.qza
	--o-visualization taxonomy.rep-seqs_XX.qzv
qiime taxa barplot	--i-table table_XX.qza
	--i-taxonomy taxonomy.rep-seqs_XX.qza
	--m-metadata-file XX.txt
	--o-visualization taxa-bar-plots_XX.qzv
qiime phylogeny align-to-tree-mafft-fasttree	--i-sequences rep-seqs_XX.qza

	--o-alignment aligned-rep-seqs_XX.qza<br>--o-masked-alignment masked-aligned-rep-seqs_XX.qza<br>--o-tree unrooted-tree_XX.qza<br>--o-rooted-tree rooted-tree_XX.qza<br>--p-n-threads 0
qiime diversity alpha-rarefaction	--i-table table_XX.qza<br>--i-phylogeny rooted-tree_XX.qza<br>--p-max-depth XXXXX<br>--m-metadata-file XX.txt<br>--o-visualization alpha-rarefaction_XX.qzv
qiime diversity core-metrics-phylogenetic	--i-phylogeny rooted-tree_XX.qza<br>--i-table table_XX.qza<br>--p-sampling-depth XXXX<br>--m-metadata-file XX.txt<br>--output-dir core-metrics-results-XX
qiime diversity beta-group-significance<br>(for bray curtis distance)	--i-distance-matrix bray_curtis_distance_matrix.qza<br>--m-metadata-file XX.txt<br>--m-metadata-column XX<br>--o-visualization bray_curtis_distance_Group.qzv<br>--p-pairwise
qiime diversity beta-group-significance<br>(for weighted unifrac distance)	--i-distance-matrix weighted_unifrac_distance_matrix.qza<br>--m-metadata-file XX.txt<br>--m-metadata-column XX<br>--o-visualization weighted_unifrac_distance_Group.qzv<br>--p-pairwise

A : QIIME2 version 2019.4 (https://docs.qiime2.org/2019.4/) was used in this study.

B : silva-132-99-nb-classifier

*The default setting of QIIME2 software, where the confidence of the taxon classification was 0.7, was used in this study.
